# Rosmarinic Acid Attenuates *Salmonella enteritidis*-Induced Inflammation via Regulating TLR9/NF-κB Signaling Pathway and Intestinal Microbiota

**DOI:** 10.3390/antiox13101265

**Published:** 2024-10-18

**Authors:** Dandan Yi, Menghui Wang, Xia Liu, Lanqian Qin, Yu Liu, Linyi Zhao, Ying Peng, Zhengmin Liang, Jiakang He

**Affiliations:** 1 College of Animal Science and Technology, Guangxi University, Nanning 530004, China; 2118402010@st.gxu.edu.cn (D.Y.); 2218302032@st.gxu.edu.cn (M.W.); 2118302028@st.gxu.edu.cn (X.L.); 2018302030@st.gxu.edu.cn (L.Q.); 2018402003@st.gxu.edu.cn (Y.L.); 2218302046@st.gxu.edu.cn (L.Z.); 2118302033@st.gxu.edu.cn (Y.P.); 2 Guangxi Key Laboratory of Animal Breeding, Disease Control and Prevention, Nanning 530004, China; 3 Guangxi Zhuang Autonomous Region Engineering Research Center of Veterinary Biologics, Nanning 530004, China

**Keywords:** Rosmarinic acid, *Salmonella enteritidis*, TLR9, NF-κB, Gut microbiota

## Abstract

*Salmonella enteritidis* (*SE*) infection disrupts the homeostasis of the intestinal microbiota, causing an intestinal inflammatory response and posing a great threat to human and animal health. The unreasonable use of antibiotics has led to an increase in the prevalence of drug-resistant *SE*, increasing the difficulty of controlling *SE*. Therefore, new drug strategies and research are urgently needed to control *SE*. Rosmarinic acid (RA) is a natural phenolic acid with various pharmacological activities, including antioxidant, anti-inflammatory and antibacterial properties. However, the protective effects and mechanism of RA on intestinal inflammation and the gut microbial disorders caused by *SE* have not been fully elucidated. In this study, RAW264.7 cells, MCECs and BALB/c mice were challenged with *SE* to assess the protective effects and mechanisms of RA. The results showed that RA enhanced the phagocytic ability of RAW264.7 cells, reduced the invasion and adhesion ability of *SE* in MCECs, and inhibited *SE*-induced inflammation in cells. Moreover, RA inhibited the activation of the NF-κB signaling pathway by upregulating TLR9 expression. Importantly, we found that RA provided protection against *SE* and increased the diversity and abundance of the intestinal microbiota in mice. Compared with infection control, RA significantly increased the abundance of *Firmicutes* and *Acidibacteria* and decreased the abundance of *Proteobacteria*, *Epsilonbacteraeota* and *Bacteroidota*. However, RA failed to alleviate *SE*-induced inflammation and lost its regulatory effects on the TLR9/NF-κB signaling pathway after destroying the gut microbiota with broad-spectrum antibiotics. These results indicated that RA attenuated *SE*-induced inflammation by regulating the TLR9/NF-κB signaling pathway and maintaining the homeostasis of the gut microbiota. Our study provides a new strategy for preventing *SE*-induced intestinal inflammation.

## 1. Introduction

*Salmonella* invasion and intracellular replication within host cells result in gastroenteritis, bacteremia, enteric fever and focal infections [1]. *Salmonella* is prone to contaminating animal foods. It is frequently detected in seafood [2], beef [3], chicken and pork [4], causes significant economic losses in animal husbandry and seriously endangers public health [5,6]. *Salmonella* invades intestinal epithelial cells, inducing an excessive inflammatory response in the gut and disrupting intestinal mucosa barrier function [7]. The virulence factor T3SS-2 of *Salmonella* enhances its colonization in the intestinal lumen by achieving chemotactic-dependent utilization of intestinal inflammation [7]. Maintaining the intestinal barrier integrity and intestinal microbiota homeostasis offers colonization resistance against *Salmonella*, limiting bacterial expansion and transmission [8]. Moreover, enhancing the antioxidant capacity of epithelial cells and inhibiting excessive inflammatory responses in the intestine contribute to the control of *Salmonella* infection [9].

Macrophages are recruited to the site of infection to exert their phagocytic function after infection with *Salmonella*. Moreover, this response has been studied intensively in epithelial cells, the target of *Salmonella* during gastrointestinal infections [10]. The ability of immune cells, particularly macrophages, to eliminate bacteria is often associated with pattern recognition receptors (PRRs), especially TLRs. TLRs activate signaling pathways that provide specific immunological responses tailored to microbes expressing PAMPs [11]. TLRs include cell surface TLRs and endosomal TLRs [12]. TLR9 is an intracellular TLR that recognizes unmethylated CpG motifs in the double-stranded DNA of bacteria. TLR9 activation amplifies the T-cell response and promotes bacterial clearance; thus, TLR9 activation may be an effective strategy for resisting pathogen entry [13]. Previous studies have proven that TLR9 is important for the host defense against *Salmonella typhimurium* invasion in mice [14]. Persistent infection with *Salmonella* leads to an inflammatory response in the body, and the NF-κB signaling pathway plays an important role in *Salmonella*-induced inflammation. TLR9 negatively regulates the NF-κB-NLRP3-IL-1β pathway and plays a vital role in defense against *Salmonella typhimurium* and the protection of the intestinal integrity. Moreover, CpG treatment induced increased TLR9 expression and enhanced the host resistance to *Salmonella* [15]. These studies indicated that activating TLR9 and inhibiting the NF-κB pathway are beneficial for the host to clear *Salmonella* and alleviate intestinal inflammatory damage.

The gut microbiota promotes the integrity of the intestinal mucosa, provides essential nutrients such as vitamins and enzymes, protects hosts from pathogen infections and regulates innate and adaptive immunity [16]. The metabolites produced by the symbiotic gut microbiota affect the host’s health through recognition by the immune system [17]. The disturbance of the gut microbiota leads to cardiac metabolism disorders, type 2 diabetes, obesity, and various chronic inflammatory diseases [18,19,20]. Moreover, the gut microbiota is crucial for resisting the colonization of exogenous microorganisms [21]. Mice harboring low-complexity gut microbiota are unable to resist *Salmonella* infection [22]. Maintaining normal gut microbiota can reduce the colonization of *Salmonella* and maintain intestinal barrier function [23].

*Salmonella enteritidis* (*SE*), a common serotype of *Salmonella*, is one of the main pathogens causing acute gastroenteritis. Currently, the main prevention and treatment drugs for *SE* infection are antibiotics, and the unreasonable use of antibiotics has led to the emergence of *SE* resistance and damage to intestinal barrier function. Residues of veterinary drugs in animal food impair public health and safety, and because of their powerful biological activity and nontoxicity and the advantage of having multiple targets, the effective monomeric components of Chinese herbal medicine are an outstanding choice for controlling *Salmonella* [24]. Rosmarinic acid (RA) is a bioactive phenolic compound commonly found in plants of the *Lamiaceae* and *Boraginaceae* families, and possesses anti-inflammatory, anticancer and antibacterial activities [25,26,27]. RA has been widely used in the beverage, food and skincare industries [28,29], provides protection against colitis and inhibits the growth of *Escherichia coli*, *Proteus mirabilis* and *Bacillus cereus* [28,30,31]. Additionally, RA has direct antibacterial and bactericidal effects on *Salmonella* in vitro [32]. However, the role of RA on *SE* infection in vivo and the regulatory mechanism of RA against *SE* are unclear.

In this study, we investigated the protective effects of RA on *Salmonella*-challenged RAW264.7 cells, MCECs and BALB/c mice, and the regulatory effects of RA on the TLR9/NF-κB signaling pathway and the gut microbiota. Overall, this study revealed that RA has a good protective effect on *SE*-induced inflammation and intestinal damage in mice by regulating the TLR9/NF-κB signaling pathway and gut microbiota. These findings indicate that RA is a mild, effective and potentially effective agent with few side effects that can be used to prevent *SE* infection.

## 2. Materials and Methods 

### 2.1. Reagents

RA was purchased from McLean Biochemical Technology Co., Ltd. (Shanghai, China); LB nutrient agar and nutrient broth were purchased from Beijing Land Bridge Co., Ltd. (Beijing, China); DMEM and FBS were purchased from Thermo Fisher Scientific (Waltham, MA, USA); RNAlater was purchased from Genstar (Guangzhou, China); FITC was purchased from Sigma-Aldrich (Shanghai, China); rabbit monoclonal antibodies against p-p65, p65, p-IκB-α, IκB-α and β-actin were purchased from Abmart (Shanghai, China); ELISA kits were purchased from Mlbio (Shanghai, China); SOD, MDA, GPT and GOT biochemical indicator detection kits were purchased from Nanjing Jiancheng Biology Co., Ltd. (Nanjing, China); and E6446 dihydrochloride was purchased from Selleck (Chongqing, China).

### 2.2. Animal 

BALB/c mice (female, 6–8 weeks, 18–22 g) were purchased from Beijing Si Bei Fu Biotechnology Co., Ltd. (Beijing, China). All mice received food and water ad libitum. The laboratory temperature was maintained at 22–24 °C, with a relative humidity of 40–50%. This study was conducted according to the recommendations of the Academy of Animal Research Guidelines and approved by the Animal Ethics Committee of Guangxi University (protocol number: GXU-2022-336). All animal experiments involving mice protocols and procedures were performed according to the protocols for the care of laboratory animals, Ministry of Science and Technology People’s Republic of China. The mice were euthanized using anesthesia.

### 2.3. Bacteria and Cell Culture

The *Salmonella enterica* (*SE*) serovar *Enteritidis* strain was isolated from chickens and maintained by the Pharmacology and Pathology Laboratory of the School of Animal Science and Technology, Guangxi University. *SE* was grown in LB broth at 37 °C and shaken at 180 rpm for 6 h, centrifuged at 3000 rpm for 10 min, and resuspended with PBS for challenge.

RAW264.7 cells and MCECs were cultured in high-glucose Dulbecco’s modified Eagle’s medium (DMEM) supplemented with 10% fetal bovine serum and penicillin streptomycin, and incubated with 5% CO_2_ at 37 °C. 

### 2.4. Minimum Inhibitory Concentration (MIC)

RA was dissolved and diluted with LB broth. Then, 100 μL of RA at different concentrations (0.0157–8 mg/mL) was added to the 96-well plate. Next, 100 μL of *SE* was added, and the final concentration of RA was 0.00785–4 mg/mL. The experiment was conducted three times in parallel.

### 2.5. CCK-8 Assay

A CCK-8 kit was used to evaluate the impact of RA on the viability of RAW 264.7 cells and MCECs. Briefly, cells (10^5^/well) in antibiotic-free medium were grown on 96-well plates at 37 °C for 12 h. Except for the control wells, the cells were treated with different concentrations of RA (0–480 μg/mL) and incubated at 37°C in 5% CO_2_ for 6 h, 12 h, 24 h or 48 h. Then, 10 μL of CCK-8 reagent was added. After incubation at 37 °C for 2 h, the optical density (OD) was determined at 450 nm via a microplate reader. The experiment was conducted six times in parallel. Survival rate data in the blank control group was compared with other groups at the same time point for statistical analysis. The percentage survival was determined using the following formula:

Viability (%) = (OD_treated cells_ − OD_control_)/(OD_control cells_ − OD_control_) × 100%

### 2.6. CFU Detection

RAW264.7 cells and MCECs (10^5^/well) were cultured in antibiotic-free medium on 24-well plates for 12 h, followed by pretreatment with different concentrations of RA (0–60 μg/mL) for 6 h. Then, *SE* was challenged with an MOI of 100, the supernatant was removed 2 h after the challenge, and the cells were washed with PBS three times to remove unattached bacteria. For the adhesion assay, the cells were incubated with 0.1% Triton X-100, and the suspensions were serially diluted with PBS and then plated on LB plates. The plates were cultured at 37°C for 16–18 h. Finally, the number of *SE* colonies that adhered to the cells was counted. For the invasion assay, the cells were washed with PBS three times after incubation with 0.1% Triton X-100 and then incubated in DMEM supplemented with penicillin and streptomycin (100 μg/mL) for 60 min. Furthermore, the cells were lysed with 1% Triton X-100, and the number of intracellular bacteria was determined via an adhesion assay. Finally, the number of *SE* colonies that invaded the cells was counted.

### 2.7. Phagocytosis Test

The cells were pretreated with RA (0–60 μg/mL) for 6 h and washed with PBS after pretreatment. Then, the cells were challenged with FITC-labeled *SE* at an MOI of 100 for 2 h, washed with PBS three times and fixed with 4% formaldehyde for 15 min. Finally, the cells were treated with DAPI for 10 min and observed using a fluorescence microscope.

### 2.8. Inflammatory Factor Detection

Cells were pretreated with RA (0–60 μg/mL) for 6 h and then challenged with *SE* (MOI: 100) for 2 h. The supernatant was collected to measure cytokine levels. The cells were lysed with TRIzol or RIPA buffer and then stored at −80 °C for RT–qPCR or Western blot detection.

### 2.9. Molecular Docking

The crystal structure of the target protein TLR9 was retrieved from the PDB database (PDB-No. 3WPF). The molecular structure of RA was obtained from the PubChem compound database. We prepared and converted the protein and ligand files into PDBQT format. Water molecules were removed from the protein structure, and hydrogen atoms were added accordingly. The grid box dimensions were determined based on the protein domain. PyMol 3.0 software was used to visualize the docking model.

### 2.10. RA Pretreatment and SE Challenge in Mice 

To evaluate the effects of RA on the survival rate of *SE*-challenged mice, the mice were randomly divided into two groups: the *SE* infection control group and the RA + *SE* group. The mice in the RA + *SE* group were orally administered RA (20 mg/ kg·bw) daily for 7 days, and the mice in the *SE* infection control group were orally administered the same dose of PBS. Then, the mice were intraperitoneally challenged with *SE* (2.5 × 10^8^ CFUs/mL, 0.2 mL) and observed for 15 days. Body weight and mortality were recorded daily.

To evaluate the effects of RA on *SE*-induced enteritis, the mice were randomly divided into four groups: the blank control group, the RA control group, the *SE* infection control group and the RA + *SE* group. Mice in the RA control group and RA + *SE* group were orally administered with RA (20 mg/ kg·bw) daily for 7 days, and mice in *SE* group and the blank control group were orally administered an equivalent volume of PBS. On the 7th day, 12 h after RA pretreatment, the mice were intraperitoneally injected with *SE* (2.5 × 10^8^ CFUs/mL, 0.2 mL). The mice were euthanized 24 h after the challenge. Serum, intestinal tissues and feces were collected for further analysis.

To analyze the effects of the gut microbiota on RA-induced protection, the mice were randomly divided into four groups: the blank control group, the *SE* infection control group, the antibiotic control group and the RA + *SE* + antibiotic group. Mice in the antibiotic control group and the RA + *SE*+ antibiotic group were orally administered with broad-spectrum antibiotics. Six hours after antibiotic treatment, mice in the RA + *SE* + antibiotics group were orally administered with RA (20 mg/ kg·bw) daily for 7 days, and the other mice were orally administered an equivalent volume of PBS. On the 7th day, 12 h after RA pretreatment, the mice were intraperitoneally injected with *SE* (2.5 × 10^8^ CFUs/mL, 0.2 mL). The mice were euthanized 24 h after the challenge. Serum, intestinal tissues and feces were collected for further analysis. Broad-spectrum antibiotics included neomycin (100 mg/L), streptomycin (50 mg/L), penicillin (100 mg/L), vancomycin (50 mg/L) and metronidazole (100 mg/L).

### 2.11. Measurement of Biochemical Indicators

The levels of glutamic oxaloacetic transaminase (GOT), glutamic pyruvic transaminase (GPT), malondialdehyde (MDA) and SOD in mouse serum were detected with a biochemical reagent kit according to the manufacturer’s instructions.

### 2.12. Measurement of Cytokine Levels

The levels of TNF-α, IL-1β and IL-6 were detected via ELISA kits according to the manufacturer’s instructions.

### 2.13. Histological Evaluation

Duodenal and colon tissues were collected, fixed in 4% paraformaldehyde and embedded in paraffin. Tissues were cut into 3 μm series slices, dewaxed in xylene, rehydrated in an ethanol gradient and then stained with hematoxylin and eosin. Histological pathology was scored according to a previously established scoring system on the basis of the degree of epithelial damage, inflammatory infiltrate in the mucosa, submucosa and muscularis/serosa, crypt damage, severity of inflammation, intestinal villus injury and damage to intestinal glands [33].

### 2.14. RT-qPCR

Total RNA was extracted from cells and intestinal tissues using a TRIzol kit according to the manufacturer’s instructions. Reverse transcription was performed with an ABM reagent kit according to the manufacturer’s instructions. The primers used for real-time PCR are listed in the Appendix A.1 (Table A1). All samples were analyzed via the Roche 96 Light Cycle 96 system (Roche, Sweden) and programmed to undergo one cycle (95 °C for 2 min) and 40 cycles (95 °C for 15 s, 60 °C for 30 s, and 72 °C for 20 s). The mRNA expression levels of the targeted genes were normalized to that of β-actin. All experiments were performed in triplicate.

### 2.15. Western Blot

Cells and intestinal tissues were lysed in RIPA lysis buffer. Protein concentrations were determined using the Bradford or bicinchoninic acid (BCA) kit. Aliquots containing 20 μg of protein were resolved by 10% sodium dodecyl sulfate-polyacrylamide gel electrophoresis (SDS-PAGE) and transferred to PVDF membranes. The membranes were blocked with 5% BSA for 2 h at room temperature before being incubated overnight with primary antibodies diluted 1:1500 in 5% BSA. The membranes were then washed five times in 1× TBST, incubated with a secondary antibody conjugated to horseradish peroxidase at a dilution of 1:3000 with BSA at 37 °C for 1 h, and processed with an enhanced chemiluminescence (ECL) kit.

### 2.16. Analysis of 16S rDNA Sequencing

Intestinal contents were collected and stored at −80 °C. Fecal genomic DNA extraction and the evaluation of DNA purity and concentration were performed by Lianchuan Biotechnology Co., Ltd. (Hangzhou, China). PCR amplification of 16S rDNA, library construction, ion S5 sequencing and bioinformatics analysis were performed on the V3-V4 domain of 16S rDNA in the gut microbiota.

### 2.17. Data Analysis

All data were analyzed uisng GraphPad Prism (version 8. 0) to test for normal distributions. For normally distributed data, one-way analysis of variance (ANOVA) followed by Tukey’s multiple comparison test was used to analyze significance. For non-normally distributed data, the Kruskal-Wallis test was used to analyze the body weight of mice, and the Mantle-Cox test was used to analyze the survival rates of mice, and the results were expressed as the mean ± standard deviation (SD); a *p*-value less than 0.05 was considered statistically significant. 

## 3. Results

### 3.1. RA Alleviated Salmonella Enteritidis (SE) -Induced Inflammatory Responses In Vitro 

**Figure 1:**
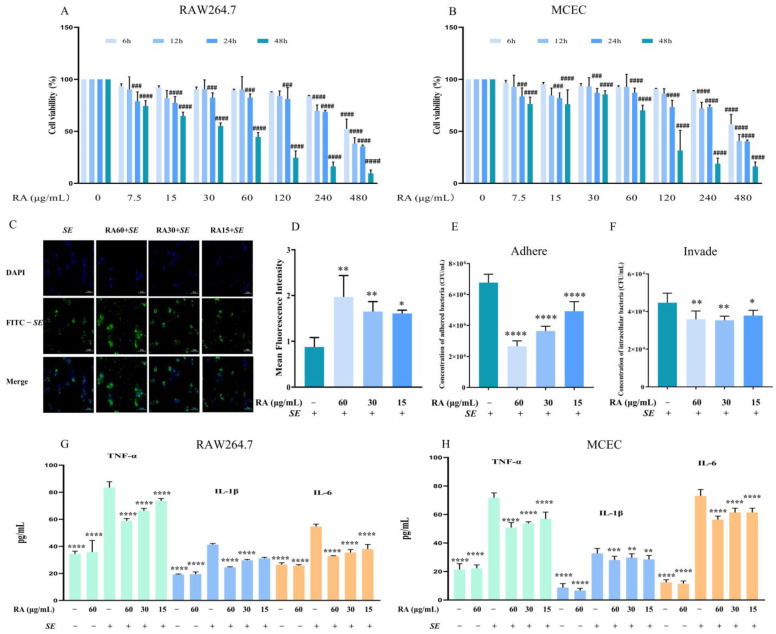
RA alleviated the cellular inflammation caused by *SE*. (**A**,**B**) Effects of RA on the viability of RAW264.7 cells and MCECs. The cells were treated with different concentrations of RA for 6 h, 12 h, 24 h, or 48 h, and a CCK-8 assay was used to detect cell viability. The cell survival rate data at the same time were obtained for statistical analysis (*n* = 6). (**C**,**D**) The fluorescence images and intensity of the phagocytic ability of RAW264.7 cells. The cells were pretreated with different concentrations of RA and challenged with FITC-labeled *SE* (MOI = 100) for 2 h (*n* = 3). (**E**) The bacteria adhering to MCECs. The cells were pretreated with different concentrations of RA, challenged with *SE* (MOI = 100) for 2 h and lysed with PBS containing 0.1% Triton X-100. The bacteria adhering to the cells were detected (*n* = 6). (**F**) The intracellular bacteria in MCECs. The cells were treated with RA and challenged with *SE* (MOI = 100) for 2 h, and then treated with DMEM containing 1% penicillin and streptomycin for 1 h. The cells were subsequently lysed with PBS containing 1% Triton X-100 and the intracellular bacteria were detected (*n* = 6). (**G**) Levels of TNF**-**α, IL**-**1β and IL**-**6 in RAW264.7 cells (*n* = 6). (**H**) Levels of TNF-α, IL-1β and IL-6 in MCECs (*n* = 6). Cells were pretreated with different concentrations of RA for 6 h, and challenged with *SE* (MOI = 100) for 2 h. Cytokine levels in the cell supernatant were detected via ELISA. Statistical analysis was performed by one-way ANOVA followed by Tukey’s multiple comparison test, and expressed as the mean ± SD, * *p* < 0.05, ** *p* < 0.01, *** *p* < 0.001, **** *p* < 0.0001 versus the *SE* infection control group, ^###^ *p* < 0.001, ^####^ *p* < 0.0001 versus the blank control group.

The MIC of RA against *SE* was 1 mg/mL (Appendix A.2 (Table A2)). Moreover, pretreatment with 60 μg/mL of RA for 6 h had no significant effect on the viability of RAW264.7 cells and MCECs. Thus, 60 μg/mL RA was used for subsequent experiments (Figure 1A,B). To investigate the effects of RA on the phagocytic ability of RAW264.7 cells and the ability of *SE* to adhere to and invade MCECs, cells were challenged with FITC-labeled *SE*. Compared with the untreated control, RA significantly enhanced the RAW264.7 cell phagocytosis of *SE* in a dose-dependent manner (Figure 1C,D). Moreover, RA significantly reduced the invasion and adhesion of *SE* in MCECs (Figure 1E,F). *SE* infection causes the excessive secretion of proinflammatory cytokines. The RA pretreatment significantly decreased the levels of TNF-α, IL-1β and IL-6 (Figure 1G,H), indicating that RA inhibited the *SE*-induced inflammatory response. Overall, these results indicated that RA promoted macrophage phagocytosis, inhibited the adhesion and invasion of *SE* and alleviated the *SE*-induced inflammatory response in vitro.

### 3.2. RA Regulated TLR9/NF-κB Signaling Pathway

**Figure 2:**
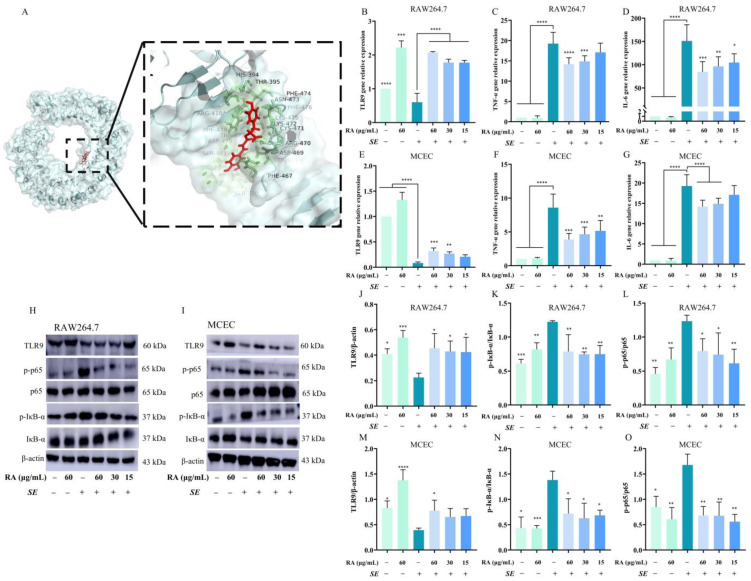
RA alleviated inflammation caused by *SE* via regulating the TLR9/NF-κB signaling pathway. Cells were pretreated with different concentrations of RA for 6 h and challenged with *SE* (MOI = 100) for 2 h; cells were collected for RT-qPCR or Western blot detection. (**A**) Molecular docking analysis of the binding mode and affinity of RA for TLR9. (**B**–**D**) The mRNA expression levels of TLR9, TNF-α and IL-6 in RAW264.7 cells (*n* = 6). (**E**–**G**) The mRNA expression levels of TLR9, TNF-α and IL-6 in MCECs (*n* = 6). (**H**–**O**) The protein expression levels of TLR9, p65, p-p65, IκB-α and p-IκB-α in RAW264.7 cells and MCECs (*n* = 3). Statistical analysis was performed by one-way ANOVA followed by Tukey’s multiple comparison test, and expressed as the mean ± SD, * *p* < 0.05, ** *p* < 0.01, *** *p* < 0.001, **** *p* < 0.0001 versus the *SE* infection control group.

TLRs and the NF-κB signaling pathway play significant roles in bacterial invasion. In this study, we evaluated the effects of RA on the expression of TLR9 and the NF-κB signaling pathway. To assess the binding energy and interaction mode between RA and TLR9, we used Python 3.13.0, Yasara 24.10.5 and DS4.0 software to determine the protein–ligand docking and binding energy for each interaction. The results showed that RA could bind to the TLR9 protein, forming hydrogen bonds with the amino acid residues HIS394, ARG470 and ASP469 (Figure 2A). In addition, the free binding energy of RA for the TLR9 protein was −8.61 kcal/mol (Appendix A.3 (Table A3)), indicating that RA could spontaneously and stably bind to TLR9. The *SE* challenge inhibited the mRNA expression of TLR9 and increased the mRNA expression of TNF-α and IL-6, and RA significantly reversed these changes (Figure 2B–G). Furthermore, we found that RA pretreatment significantly increased the protein expression level of TLR9 and inhibited the protein expression levels of p-p65 and p-IκB-α compared to the *SE* infection control group (Figure 2H–O). Collectively, these results revealed that RA regulated TLR9 and inhibited the phosphorylation of p65 and IκB-α to limit the activation of the NF-κB signaling pathway against *SE* infection.

### 3.3. RA Inhibited NF-κB Signaling Pathway by Upregulating TLR9

**Figure 3:**
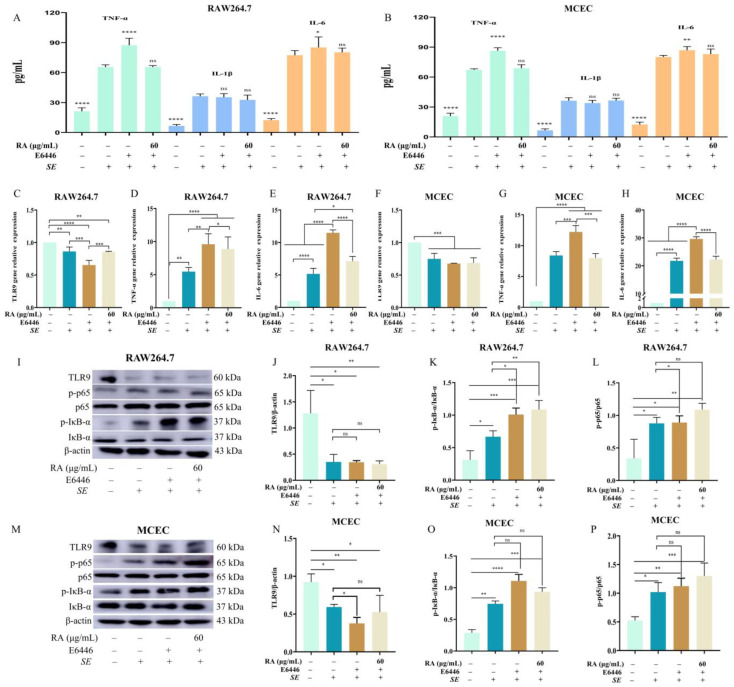
RA inhibited the activation of NF-κB signaling pathway by upregulating TLR9. The cells were pretreated with 10 μM E6446 (TLR9 inhibitor dihydrochloride) for 2 h, and co-incubated with RA for 6 h and then challenged with *SE* (MOI = 100) for 2 h. The cell supernatants were collected to detect cytokines, and the cells were collected for RT–qPCR or Western blot detection. (**A**,**B**) Levels of proinflammatory cytokines in RAW264.7 cells and MCECs (*n* = 6). (**C**–**E**) The mRNA expression levels of TLR9, TNF-α and IL-6 in RAW264.7 cells (*n* = 6). (**F**–**H**) The mRNA expression levels of TLR9, TNF-α and IL-6 in MCECs (*n* = 6). (**I**–**L**) The protein expression levels of TLR9, p65, p-p65, IκB-α and p-IκB-α in RAW264.7 cells (*n* = 3). (**M**–**P**) The protein expression levels of TLR9, p65, p-p65, IκB-α and p-IκB-α in MCECs (*n* = 3). For (**A**,**B**), statistical analysis was performed by one-way ANOVA followed by Tukey’s multiple comparison test, and expressed as the mean ± SD, ^ns^ *p* > 0.05, * *p* < 0.05, ** *p* < 0.01, *** *p* < 0.001, **** *p* < 0.0001 versus the *SE* infection control group. For (**C**–**P**), statistical analysis was performed by one-way ANOVA followed by Tukey’s multiple comparison test, and expressed as the mean ± SD, ^ns^ *p* > 0.05, * *p* < 0.05, ** *p* < 0.01, *** *p* < 0.001, **** *p* < 0.0001.

To confirm the relationship between TLR9 and the NF-κB signaling pathway in RA-mediated anti-inflammatory effects, the cells were pretreated with the TLR9 inhibitor E664 dihydrochloride. In the presence of E664 dihydrochloride, *SE* infection significantly increased the levels of TNF-α, IL-1β and IL-6 and reduced the mRNA expression level of TLR9. After pretreatment with E664 dihydrochloride, RA pretreatment failed to reverse these effects (Figure 3A–H). The protein expression levels of p-p65 and p-IκB-α in the inhibitor-treated group were significantly greater than those in the blank group, indicating that the suppression of TLR9 led to the activation of the NF-κB signaling pathway and caused the loss of the regulatory effect of RA on the NF-κB signaling pathway (Figure 3I–P). Overall, these results suggested that RA alleviated the *SE*-induced inflammatory response by promoting TLR9 expression to inhibit the NF-κB signaling pathway.

### 3.4. RA Provided Protection against SE in Mice

**Figure 4:**
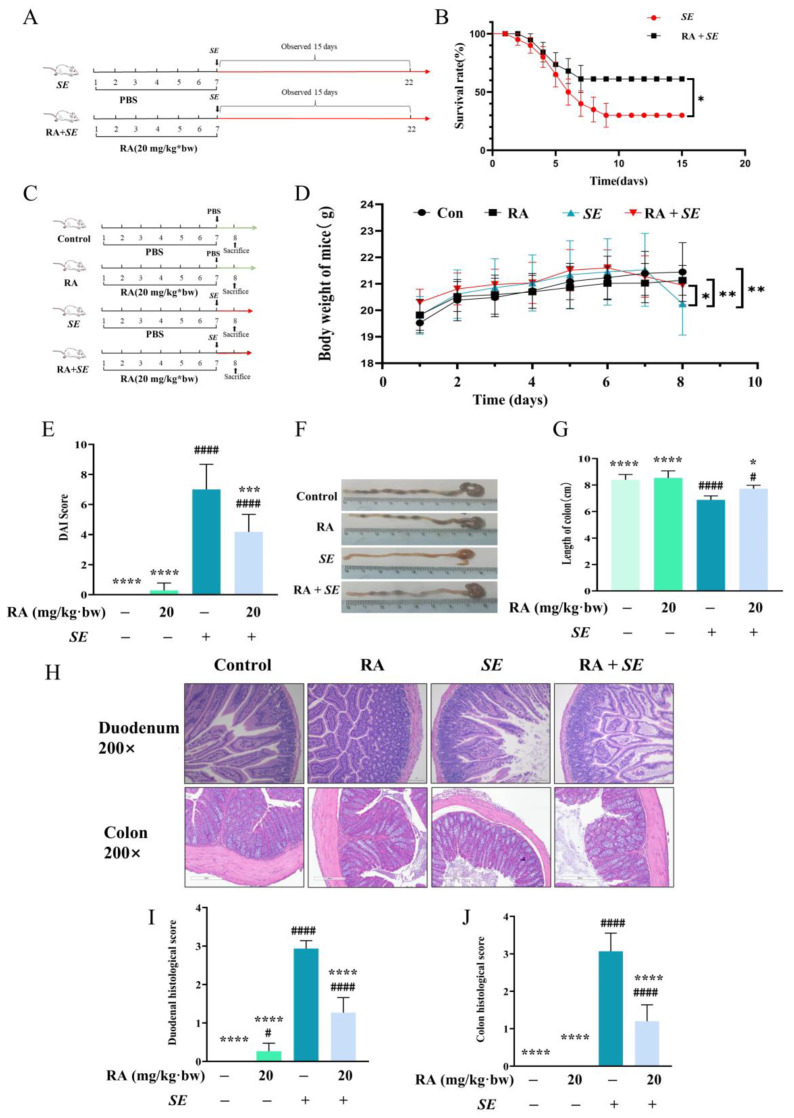
Protective effects of RA on *SE*-challenged mice. (**A**) Experimental design for evaluating the effect of RA on the survival rate of mice challenged with *SE*. Mice were orally pretreated with RA (20 mg/kg**·**bw) for 7 days and then intraperitoneally injected with 0.2 mL of *SE* at a concentration of 2.5 × 10^8^ CFUs/mL. The mortality of mice was recorded. (**B**) Survival rate of the mice (*n* = 20). (**C**) Experimental design for testing the protective effect of RA against *SE*. The treatment and challenge of the mice were the same as those in (**A**), and samples were collected 24 h after challenge. (**D**) Changes in the body weights of the mice (*n* = 10). (**E**) Disease activity index (*n* = 10). (**F**,**G**) Measurement of colon length (*n* = 6). (**H**) The histopathological changes in duodenum and colon (*n* = 3). (**I**) Duodenum histopathological score (*n* = 3). (**J**) Colon histopathological score (*n* = 3). For (**B**–**D**), statistical analysis was performed by Mantle-Cox test and Kruskal-Wallis test, respectively, and *SE* infection control group was compared with other groups. In (**G**–**J**), statistical analysis was performed by one-way ANOVA followed by Tukey’s multiple comparison test, and expressed as the mean ± (SD), * *p* < 0.05, ** *p* < 0.01, *** *p* < 0.001, **** *p* < 0.0001 versus the *SE* infection control group, ^#^ *p* < 0.05, ^####^ *p* < 0.0001 versus the blank control group.

**Figure A1:**
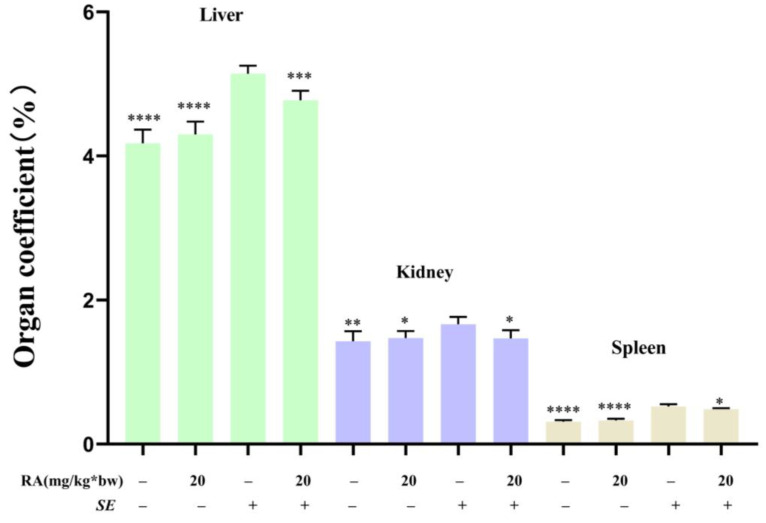
The protective effect of RA on *SE*-infected mice. Organ indices of the liver, kidney and spleen (*n* = 6). Statistical analysis was performed by one-way ANOVA followed by Tukey’s multiple comparison test, and expressed as the mean ± SD, * *p* < 0.05, ** *p* < 0.01, *** *p* < 0.001, **** *p* < 0.0001 versus the *SE* infection control group.

To investigate the protective effect and mechanism of RA on *SE* infection in vivo, the mice were orally treated with RA for 7 days. We found that RA reduced the mortality rate caused by *SE* (Figure 4A,B). Moreover, compared with the *SE* infection control group, RA significantly alleviated body weight loss, decreased the DAI, increased the length of the colon (Figure 4C–G) and decreased the organ indices of the liver and other organs (Appendix B.1, Figure A1). Consistently, we found that *SE* infection led to the enlargement of the intestinal cavity, damage to and detachment of the duodenal villi, reduction in colonic glands and thinning of the muscular layer (Figure 4H). Moreover, the RA pretreatment significantly improved the pathological damage to the duodenum and colon (Figure 4I,J). These results indicate that RA provided protection against *SE* in mice.

### 3.5. RA Alleviated SE-Induced Intestinal Inflammation and Oxidative Damage

**Figure 5:**
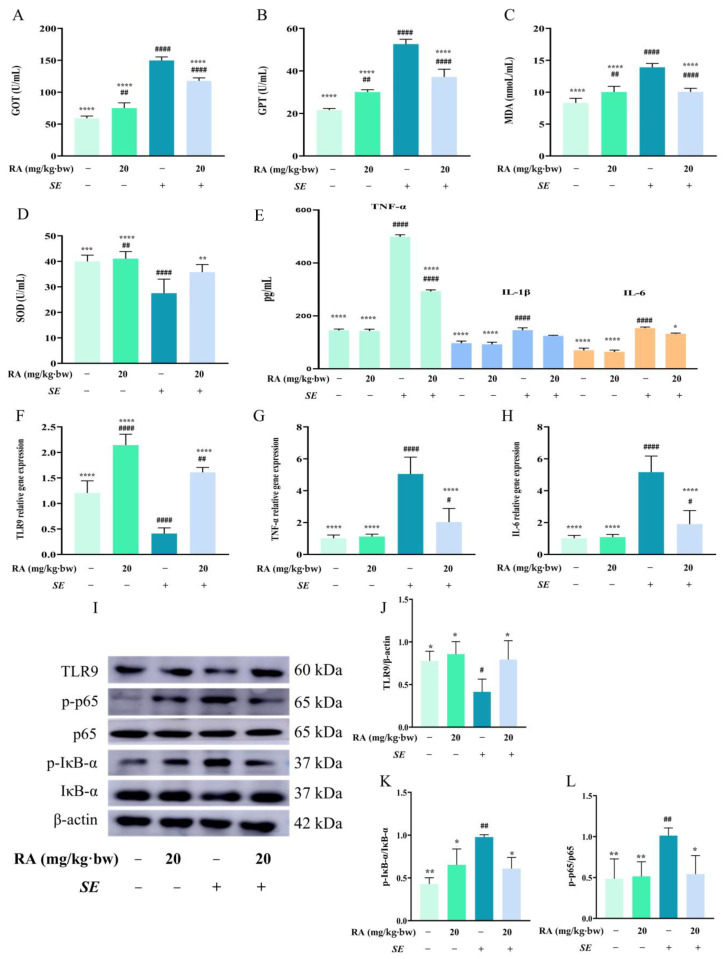
RA alleviated *SE*-induced intestinal inflammation by regulating TLR9 and inhibiting the activation of the NF-κB signaling pathway. The mice were orally pretreated with RA (20 mg/kg**·**bw) for 7 days, 0.2 mL of *SE* at a concentration of 2.5 × 10^8^ CFUs/mL was intraperitoneally injected, and samples were collected 24 h after challenge. (**A**–**D**) Serum levels of GOT, GPT, MDA and SOD (*n* = 6). (**E**) Levels of TNF-α, IL-1β and IL-6 in the serum (*n* = 6) (**F**–**H**). The mRNA expression levels of TLR9, TNF-α and IL-6 in colon tissues (*n* = 6). (**I**–**L**) The protein expression levels of TLR9, p65, p-p65, IκB-α and p-IκB-α in colon tissues (*n* = 3). Statistical analysis was performed by one-way ANOVA followed by Tukey’s multiple comparison test, and expressed as the mean ± SD, * *p* < 0.05, ** *p* < 0.01, *** *p* < 0.001, **** *p* < 0.0001 versus the *SE* infection control group, ^#^ *p* < 0.05, ^##^ *p* < 0.01, ^####^ *p* < 0.0001 versus the blank control group.

Excessive inflammation and oxidative stress cause damage to organs and tissues. In this study, we found that *SE* infection caused a significant increase in the levels of GOT, GPT and MDA in serum, and led to a significant decrease in the SOD level in serum compared with the blank control group, suggesting *SE* infection led to oxidative damage, but RA significantly decreased the production of GOT, GPT and MDA, and significantly increased the SOD production compared with that in the *SE* infection control group, indicating that RA pretreatment may alleviate oxidative damage caused by *SE* infection in mice (Figure 5A–D). Moreover, *SE* infection significantly increased the production of TNF-α, IL-1β and IL-6 and inhibited TLR9 expression compared with the blank control group; however, the RA pretreatment alleviated the overproduction of inflammatory cytokines and the suppression of TLR9 compared with the *SE* infection control group (Figure 5E–H). Moreover, in comparison with the blank control group, *SE* infection significantly increased the protein expression levels of p-p65 and p-IκB-α, while RA downregulated *SE*-caused p-p65 and p-IκB-α expression (Figure 5I), which was consistent with the findings of the cell experiments. Collectively, these results indicated that RA improved *SE*-induced inflammatory and oxidative damage in mice, which may be associated with upregulating TLR9 and inhibiting the NF-κB signaling pathway.

### 3.6. RA Improved SE-Caused Intestinal Microbiota Disorder

**Figure 6:**
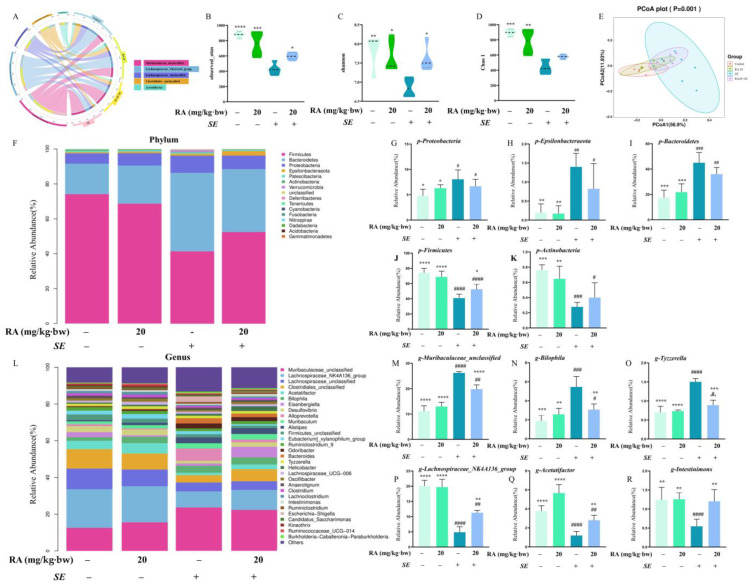
RA regulated the gut microbiota in *SE*-challenged mice (*n* = 6). The mice were orally pretreated with RA (20 mg/kg·bw) for 7 days, then intraperitoneally injected with 0.2 mL of *SE* at a concentration of 2.5 × 10^8^ CFUs/mL. (**A**) Circos diagram. (**B**) Alpha diversity analysis: observed OTUs. (**C**) Alpha diversity analysis: Shannon index. (**D**) Alpha diversity analysis: Chao 1. (**E**) β-diversity analysis: PCoA. (**F**) Relative abundance of the gut microbiota at the genus level. (**G**–**K**) Relative abundance of *p__Proteobacteria*, *p__Epsilonbacteraeota*, *p__Bacteroidota*, *p__Firmicutes* and *p__Actinobacteria*. (**L**) Relative abundance of the gut microbiota at the genus level. (**M**–**R**) Relative abundance of g__*Muribaculaceae*_unclassified, g__*Bilophila*, g__*Tyzzerella*, g__*Lachnospiraceae_NK4A136*_group, g__*Acetatifactor* and g__*Intestinimonas*. Statistical analysis was performed by one-way ANOVA followed by Tukey’s multiple comparison test, and expressed as the mean ± SD, * *p* < 0.05, ** *p* < 0.01, *** *p* < 0.001, **** *p* < 0.0001 versus the *SE* infection control group, ^#^ *p* < 0.05, ^##^ *p* < 0.01, ^###^ *p* < 0.001, ^####^ *p* < 0.0001 versus the blank control group.

**Figure A2:**
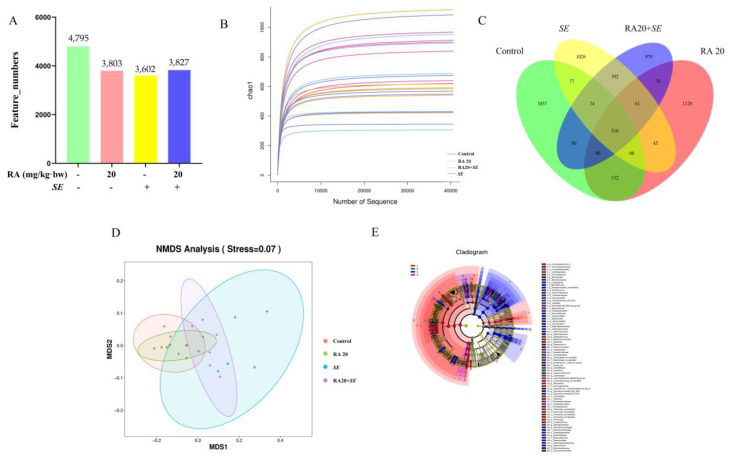
RA regulated gut microbiota in *SE*-infected mice. (**A**): Feature numbers; (**B**): rare curve of the OTU level; (**C**): Venn diagram; (**D**): non-metric multi-dimensional scaling analysis; (**E**): linear discriminant analysis effect size.

**Figure A3:**
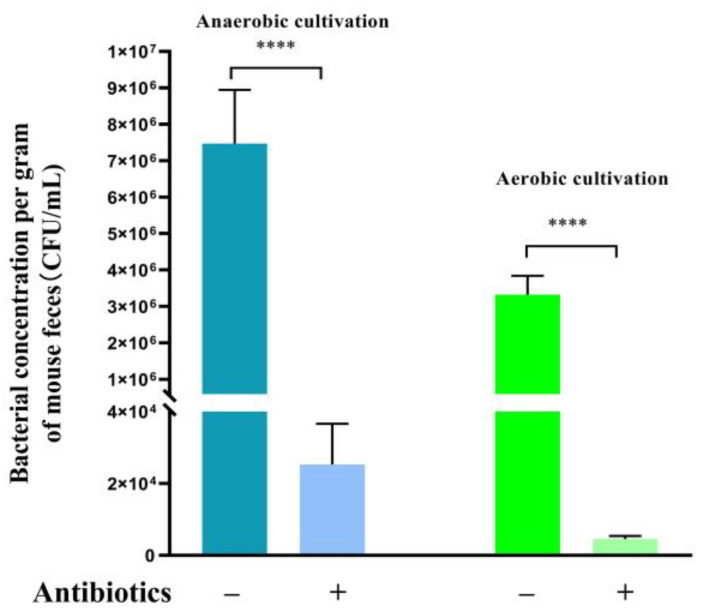
Broad-spectrum antibiotic pretreatment significantly reduced the number of intestinal bacteria in mice. The broad-spectrum antibiotics used included neomycin (100 mg/L), streptomycin (50 mg/L), penicillin (100 mg/L), vancomycin (50 mg/L), and metronidazole (100 mg/L). The mice were orally administered with 0.5 mL of broad-spectrum antibiotics by gavage for 7 d (*n* = 6). After 7 d, 10 g of mouse feces was collected under sterile conditions. The feces were diluted with 1 g/mL sterile water and cultured anaerobically and aerobically on LB medium via the dilution coating plate method. The bacterial colonies on the plate were counted after 12 h at 37 °C. Student’s *t*-test was used for statistical analysis. **** for *p* < 0.0001 versus the blank control group.

The gut microbiota plays a crucial role in maintaining intestinal homeostasis. We analyzed the composition and structure of the gut microbiota to investigate the effect of RA on the gut microbiota of mice. A total of 16,027 significant features were obtained for the 16S rRNA genes of the four bacterial groups (Figure A2A). The rarefaction curve of the OTUs of the mouse gut bacterial community tended to flatten, and the sequencing depth basically covered most of the microbial species in the sample (Figure A3B). A Venn diagram of the overlapping and shared OTU data revealed that 766 OTUs in the RA pretreatment group were shared with those in the control group, and 733 OTUs in the infection control group were shared with those in the control group (Figure A3C). A Circos diagram revealed that after the RA pretreatment, *Lachnospiraceae_NK4A136* dominated the intestinal microbiota in the mice at the genus level (Figure 6A). According to the α-diversity analysis, the species abundance of the gut microbiota in the *SE* infection control group was significantly lower than that in the control group, suggesting that *SE* could decrease the species diversity of the intestinal microbiota in mice. Compared with that in the *SE* infection control group, the α-diversity of the gut microbiota in the RA pretreatment group was significantly greater, indicating that RA had an effect on the abundance of bacterial species in the gut microbiota in the context of *SE*-induced intestinal inflammation (Figure 6B–D). A β-diversity analysis was performed via a principal coordinate analysis and non-metric multidimensional scaling analysis to explore the differences in the microbiota composition between the groups (Figure 6E and Appendix B.2, Figure A2). Clearly, a marked separation between the *SE* infection control group and the blank control group was observed, indicating that the microbiota composition was altered after modeling with *SE*. There was no overlap between the *SE* infection control group and the RA pretreatment group, indicating that RA altered the gut microbiota structure in mice infected with *SE*. These results suggested that the gut microbiota of the mice was uniquely altered after the RA supplementation. A taxonomic analysis at the phylum level revealed that *SE* infection caused an increase in the abundance of *Proteobacteria*, *Epsilonbacteraeota* and *Bacteroidota* as well as a decrease in the abundance of *Fiemicutes* and *Acidibacteria*; the RA pretreatment reversed this trend (Figure 6F–K). A taxonomic analysis at the genus level revealed that *SE* infection significantly increased the abundance of *Muribaculaceae*, *Bilophila* and *Tyzzerella* but decreased the abundance of *Lachnospiraceae_NK4A136*, *Acetatifactor* and *Intestinimonas*, and RA reversed the changes in the gut microbiota caused by *SE* (Figure 6L–R). The use of the linear discriminant analysis effect size (LEfSe) to display the microbial characteristics is the most likely to explain the differences between categories and to reveal the taxonomic groups with the greatest differences in abundance between different groups (Figure A3E). These results indicated that the RA pretreatment increased the abundance and diversity of the gut microbiota, upregulated the abundance of *Lachnospiraceae_NK4A136*, *Acetatifactor* and *Intestinimonas*, and reduced the abundance of *Muribaculaceae*, *Bilophila* and *Tyzzerella* at the genus level to improve the intestinal microbiota disorders caused by *SE*.

### 3.7. RA Failed to Provide Protection in Pseudo Germ-Free Mice

**Figure 7:**
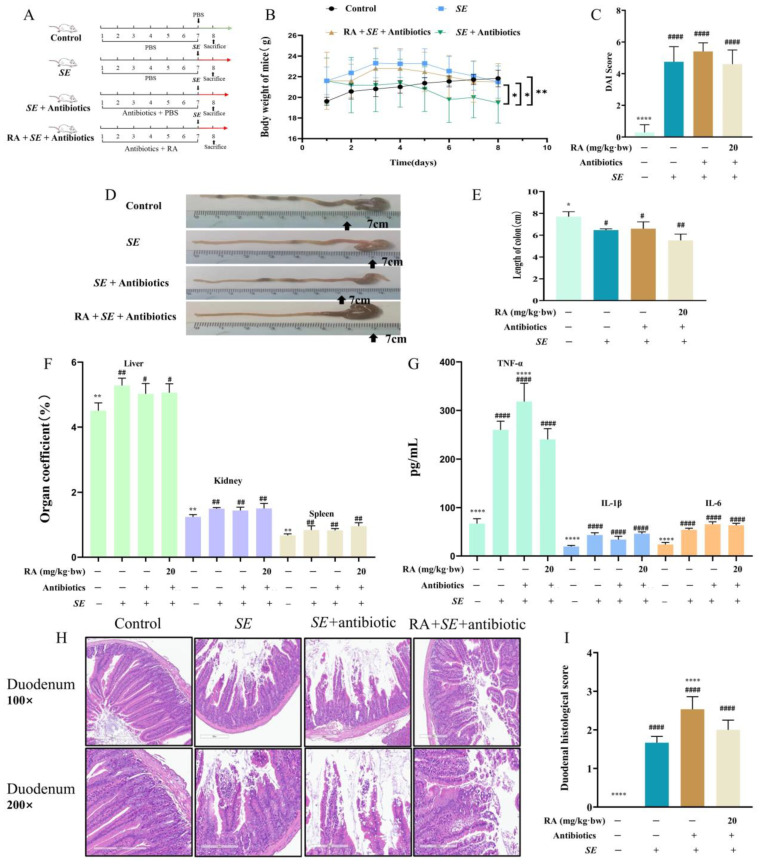
RA lost its protective effect against *SE* after clearance of the microbiota. (**A**) Experimental design in mice. The mice were orally pretreated with RA (20 mg/kg·bw) or antibiotics for 7 days, 0.2 mL of *SE* at a concentration of 2.5 × 10^8^ CFUs/mL was intraperitoneally injected, and samples were collected 24 h after challenge (*n* = 10). (**B**) Changes in body weight (*n* = 10). (**C**) Disease activity index (*n* = 10). (**D**,**E**) Measurement of colon length (*n* = 10). (**F**) Organ indices of the liver, kidney and spleen (*n* = 10). (**G**) Levels of TNF-α, IL-1β and IL-6 in the serum (*n* = 6). (**H**) Histopathological changes in the duodenum (*n* = 3). (**I**) Duodenal histopathological score (*n* = 3). For (**B**), statistical analysis was performed using Kruskal-Wallis test, and the *SE* infection control group was compared with other groups. For (**C**–**I**), statistical analysis was performed by one-way ANOVA followed by Tukey’s multiple comparison test, and expressed as the mean ± SD, * *p* < 0.05, ** *p* < 0.01, **** *p* < 0.0001 versus the *SE* infection control group, ^#^ *p* < 0.05, ^##^ *p* < 0.01, ^####^ *p* < 0.0001 versus the blank control group.

To verify the relationship between the RA-induced protection and the gut microbiota. We use of broad-spectrum antibiotics cleared the gut microbiota of the mice (Appendix B.3, Figure A3). In this study, we found that the elimination of the intestinal microbiota aggravated *SE* infection, and the mice without normal gut microbiota showed significant weight loss, increased DAI scores, intestinal enlargement and an increased organ coefficient of the liver and other organs (Figure 7A–F); the RA pretreatment did not reverse these changes. Moreover, the mice treated with broad-spectrum antibiotics presented a high level of TNF-α in serum after challenge with *SE*; RA did not alleviate the increase in TNF-α (Figure 7G). The pathological results of the duodenum showed that RA did not improve the *SE*-induced intestinal enlargement or intestinal villus damage in the duodenum (Figure 7H,I). These results suggested that the protective effect of RA against *SE* infection depends on the gut microbiota.

### 3.8. The Regulation of TLR9/NF-κB Signaling Pathway by RA Was Dependent on Gut Microbiota

**Figure 8:**
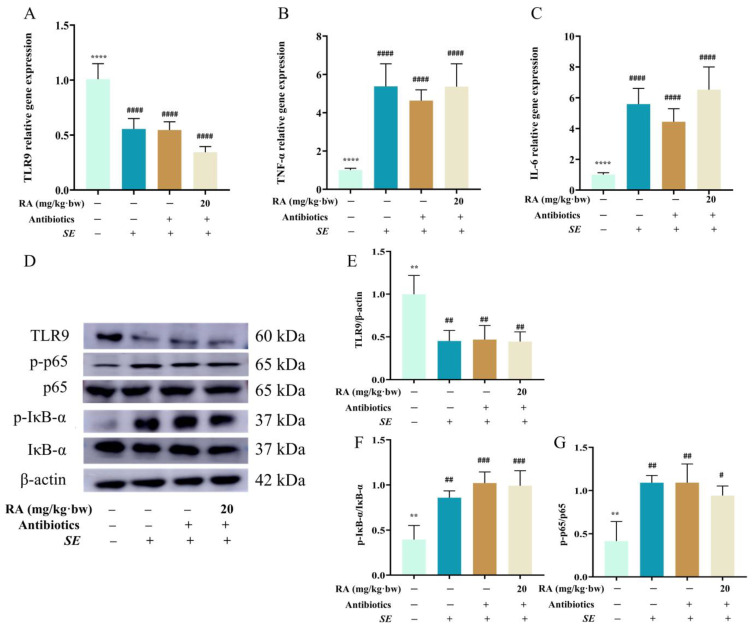
RA failed to inhibit NF-κB signaling pathway by regulating TLR9 after clearance of the microbiota. The mice were orally pretreated with RA (20 mg/ kg·bw) and antibiotics for 7 days and then intraperitoneally injected with 0.2 mL of *SE* at a concentration of 2.5 × 10^8^ CFUs/mL. Samples were collected 24 h after challenge. (**A**–**C**) The mRNA expression levels of TLR9, TNF-α and IL-6 in colon tissues (*n* = 6). (**D**–**G**) The protein expression levels of TLR9, p65, p-p65, IκB-α and p-IκB-α in colon tissues (*n* = 3). Statistical analysis was performed by one-way ANOVA followed by Tukey’s multiple comparison test, and expressed as the mean ± SD, ** *p* < 0.01, **** *p* < 0.0001 versus the *SE* infection control group, ^#^ *p* < 0.05, ^##^ *p* < 0.01, ^###^ *p* < 0.001, ^####^ *p* < 0.0001 versus the blank control group.

To determine the relationship between the TLR9/NF-κB signaling pathway and gut microbiota regulated by RA, this study detected the expression of the TLR9/NF-κB signaling pathway in pseudo sterile mice. The *SE* challenge significantly decreased the mRNA expression of TLR9, but significantly increased the mRNA expression of TNF-α and IL-6 (Figure 8A–C). Moreover, the *SE* challenge significantly decreased the protein expression of TLR9 and significantly increased the protein expression of p-p65 and p-IκB-α, but RA did not reverse these effects (Figure 8D–G). These results indicated that the loss of the regulatory effect of RA on TLR9 led to the increased activation of the NF-κB signaling pathway and exacerbated intestinal inflammation in the pseudo sterile mice, which suggested that the gut microbiota is important for the protective effect of RA on *SE* infection.

## 4. Discussion 

*Salmonella enteritidis* (*SE*) is a zoonotic pathogen that poses a great threat to human and animal health, causing significant economic losses to the livestock industry [28]. In modern society, the excessive intake of refined grains and insufficient intake of dietary fiber damage the gut microbiota, leading to a decrease in resistance to intestinal pathogen infections [34,35]. Antibiotics are often used to control *SE* infection, but their excessive use leads to the production of drug-resistant *SE*. Additionally, the excessive use of antibiotics disturbs the gut microbiota and damages the intestinal barrier [36]. Moreover, the inflammation induced by *Salmonella typhimurium* accelerated the transfer of plasmids and bacteriophages, promoting the spread of antibiotic resistance [37]. Therefore, there is an urgent need to find new drug strategies that can reduce bacterial resistance and prevent *SE* infection.

The control of inflammation and balance of the gut microbiota balance is important for resisting *SE* infection. The effective active ingredients of natural plants exhibit anti-inflammatory and antibacterial effects as well as low toxicity, and maintain the balance of the gut microbiota. Importantly, they are not prone to drug resistance. Like Ganoderma lucidum polysaccharide [38] and Astragalus polysaccharide [39], they are candidate drugs for limiting inflammation and regulating the gut microbiota caused by *SE*. RA, a compound derived from plants in the *Lamiaceae* family, has anti-inflammatory and antioxidant effects. However, its protective effects and mechanisms against *SE* challenge in vitro and in vivo are not yet clear. In this study, we found that the MIC of RA against *SE* was 1 mg/mL, which was consistent with the findings of a previous study [32]_._ Intestinal epithelial cells are the main target cells for *SE* infection, and *SE* invades intestinal epithelial cells and causes systemic infection and inflammatory reactions [40]. During infection, macrophages strongly phagocytose *SE* to reduce its adhesion and invasion to intestinal epithelial cells. Furthermore, we found that RA enhanced the ability of RAW264.7 cells to phagocytose *SE*, reduced the ability of *SE* to adhere and invade MCECs and alleviated the *SE*-induced overproduction of TNF-α, IL-1β and IL-6. Our study revealed that RA offered protection against *SE* in vivo. We found that RA reduced the mortality rate of the *SE*-challenged mice, significantly alleviated the *SE*-induced decreases in body weight and DAI, increased the organ index and shortened the colon. Additionally, the RA pretreatment significantly improved the pathological changes in the duodenum and colon in the *SE*-infected mice. Previous studies have shown that *SE* infection significantly decreased the SOD and GSH activities in Caco-_2_ cells and mice, which was consistent with our findings [9,41]. These alterations may be attributed to the active defense against the oxidative damage to intestinal epithelial cells after *Salmonella* infection [42]. Additionally, our data revealed that *SE* infection increased the TNF-α, IL-1β and IL-6 and MDA levels. MDA, a sensitive index assessing the degree of oxidative injury, was linked to chronic inflammation in the gut [43]. High levels of TNF-α, IL-1β and IL-6 and MDA in the *SE*-stimulated mice indicated that *SE* infection caused an imbalance in the redox status, resulting in oxidative stress and inflammatory responses. However, the mice pretreated with RA displayed a remarkable increase in SOD activity in serum, and a notable reduction in the levels of TNF-α, IL-1β and IL-6 and MDA in the serum, indicating that RA could enhance the antioxidant and anti-inflammatory ability to provide protection against *SE*.

TLRs are pattern recognition receptors that recognize a broad variety of structurally conserved molecules derived from microbes [44]. TLRs play a crucial role in preventing host invasion in pathogenic infections, and TLR2, TLR4 and TLR9 are involved in the defense against *Salmonella* in vivo [45]. TLR9 is vital for resistance to *Salmonella* infection, and its absence leads to severe *Salmonella* hepatitis, a high bacterial load, sustained inflammation and tissue damage [14,15,46]. In our study, we discovered that RA and TLR9 had multiple binding sites, and that RA bound well to the TLR9 protein, forming hydrogen bonds with the amino acid residues HIS394, ARG470 and ASP469. Consistently, we found that the RA pretreatment significantly upregulated the mRNA and protein expression of TLR9. After blocking TLR9 expression, RA failed to protect against *SE* infection, suggesting that the protection induced by RA was dependent on TLR9, which was consistent with the findings of a previous study, showing that TLR9 negatively regulates the NF-κB signaling pathway to alleviate IEC inflammation caused by *Salmonella typhimurium* [15]. The NF-κB signaling pathway is activated by various stimuli to regulate the inflammatory response [47]. The phosphorylation and nuclear translocation of p65 and IκB-α can activate the NF-κB signaling pathway, which plays a crucial role in regulating TNF-α, IL-1β and IL-6 [48]. Our study revealed that RA significantly inhibited the NF-κB signaling pathway to decrease the expression of TNF-α, IL-1β and IL-6, exerting a protective effect on *Salmonella*-induced intestinal and cellular inflammation. However, TLR9 suppression led to the loss of the ability of RA to inhibit the secretion of proinflammatory cytokines and the phosphorylation of p65 and IκB-α in vitro. Overall, our study suggested that RA inhibited the activation of the NF-κB signaling pathway by increasing TLR9 expression to alleviate *SE*-induced intestinal inflammation.

The gut is colonized by a large and diverse microbiota, and the microbiota composition is vital to the intestinal barrier [49]. High levels of fiber, unsaturated fatty acids and polyphenols in food are beneficial for physical health [50]. As a polyphenolic acid compound with medicinal and edible homology, RA is an effective active ingredient in plants rich in dietary fiber [51], and it has a protective effect on the gut microbiota [52]. The invasion of exogenous microorganisms leads to a decrease in the microbial diversity of the intestinal flora. Our study revealed that *SE* infection resulted in a decrease in the abundance and diversity of the gut microbiota in mice, whereas RA effectively increased the abundance and diversity of the gut microbiota. In the present study, the RA pretreatment increased the abundance of *Firmicutes* and *Acidibacteria* and decreased the abundance of *Proteobacteria*, *Epsilonbacteraeota* and *Bacteroidota* at the phylum level. *Lachnospiraceae_NK4A136* is a representative bacterium that produces butyric acid and contributes to maintaining intestinal barrier function [53]. After the RA pretreatment, *Lachnospiraceae_NK4A136* dominated the mouse gut microbiota at the genus level, suggesting that RA alleviated *Salmonella*-induced intestinal damage and maintained intestinal health through *Lachnospiraceae_NK4A136*. The long-term use of broad-spectrum antibiotics can greatly interfere with or eliminate gut microbiota, and thus weaken the resistance of the gut microbiota to colonization by exogenous pathogens [54]. After the clearance of the gut microbiota, the susceptibility of the intestine to *SE* increased [55]. Therefore, the gut microbiota plays an extremely important role in resisting *SE* infection. When the intestinal microbiota was cleared with broad-spectrum antibiotics, RA failed to alleviate inflammation and duodenum damage. Furthermore, RA also failed to regulate the expression of p-p65, p-IκB-α, proinflammatory cytokines and TLR9 in the *SE*-challenged mice, revealing that the gut microbiota played an important role in the protective effect of RA. The gut microbiota is a prerequisite for resisting exogenous pathogen infection; however, further research is needed on how RA upregulates TLR9 to inhibit the activation of the NF-κB signaling pathway through the gut microbiota to resist *SE* infection, and whether it directly affects the metabolites of the gut microbiota.

## 5. Conclusions

In summary, our study demonstrated that RA alleviated *SE*-induced inflammation by upregulating TLR9 and inhibiting the NF-κB signaling pathway, which is closely related to maintaining gut microbiota homeostasis in mice. Supplementing with RA may be a preventive strategy to alleviate the damage caused by *SE* in humans and animals. Our study provided theoretical support for the application and development of RA in the prevention and treatment of *SE* infection.

## Data Availability

The datasets used and analyzed during the current study are available from the corresponding author upon reasonable request.

## Abbreviations

TLR9: toll-like receptor-9; NF-κB, nuclear factor kappa-light-chain-enhancer of activated B cells; *SE* (used in the chart), *Salmonella enterica*; GOT, glutamic oxaloacetic transaminase; GPT, glutamic-pyruvic transaminase; IL-1β, interleukin-1β; IL-6, interleukin-6; LB, Luria-Bertani culture medium; MDA, malondialdehyde; p-p65, phosphorylated p65; p-IκB-α, phosphorylated IκB-α; RA, rosmarinic acid; SOD, superoxide dismutase; TNF-α, tumour necrosis factor α; PAMPs, pathogen-associated molecular patterns; E6446 (used in the chart), E6446 dihydrochloride; MIC, minimum inhibitory concentration; BSA, bovine serum albumin; 20 mg/kg·bw, 20 mg/ kg·body weight.

## Appendix A

### Appendix A.1

**Table A1:** Primers for RT-qPCR.

Target Gene	Forward (5′–3′)	Reverse (5′–3′)	Genbank Number
TNF-α	CGCTGAGGTCAATCTGC	GGCTGGGTAGAGAATGGA	XM_042684006.1
IL-6	ACAGAAGGAGTGGCTAAGGA	AGGCATAACGCACTAGGTTT	XM_032905335.1
TLR9	CAGGAGCGGTGAAGGTG	GAAGGGAGGTCAGATTGGC	NM_031178.2
β-actin	CCTCACTGTCCACCTTCC	GGGTGTAAAACGCAGCTC	XM_058569360.1

### Appendix A.2

The minimum inhibitory concentration of RA against *SE*. The minimum inhibitory concentration of RA against *SE* is 1 mg/mL.

**Table A2:** The minimum inhibitory concentration of RA against *SE*.

Drug Concentration (mg/mL)	4.00	2.00	1.00	0.500	0.250	0.125	0.0625	0.0313	0.0157	0.00785	Positive Control	Negative Control
1	−	−	−	+	+	+	+	+	+	+	+	−
2	−	−	−	+	+	+	+	+	+	+	+	−
3	−	−	−	+	+	+	+	+	+	+	+	−

+ represents positive, indicating the presence of bacterial precipitation, − represents negative, and no bacterial precipitation appears.

### Appendix A.3

The free binding energy of RA and the TLR9 protein is −8.6100 kcal/mL.

**Table A3:** The free binding energy of RA and TLR9 protein.

Run	Bind. Energy [kcal/mol]	Dissoc. Constant [pM]	Contacting Receptor Residues
001	000008.6100	00000000488412.0000	HIS A 394 THR A 395 ARG A 418 PHE A 419 PHE A 467 ASP A 469 ARG A 470 CYS A 471 LYS A 472 ASN A 473 LYS A 475 THR A 477 ASP A 479 SER A 481 ARG A 482 SER A 503 SER A 505 HIS A 506 ASP A 528
002	000008.6100	00000000488412.0000	HIS A 394 THR A 395 ARG A 418 PHE A 419 PHE A 467 ASP A 469 ARG A 470 CYS A 471 LYS A 472 ASN A 473 LYS A 475 THR A 477 ASP A 479 SER A 481 ARG A 482 SER A 503 SER A 505 HIS A 506 ASP A 528
003	000008.4100	00000000684523.3125	ARG A 418 PHE A 419 PHE A 467 ASP A 469 CYS A 471 LYS A 472 ASN A 473 LYS A 475 THR A 477 ASP A 479 SER A 481 SER A 503 SER A 505 ASP A 528 SER A 530
004	000008.4100	00000000684523.3125	ARG A 418 PHE A 419 PHE A 467 ASP A 469 CYS A 471 LYS A 472 ASN A 473 LYS A 475 THR A 477 ASP A 479 SER A 481 SER A 503 SER A 505 ASP A 528 SER A 530
005	000008.3810	00000000718862.0000	HIS A 394 THR A 395 HIS A 397 ARG A 418 PHE A 419 PHE A 467 ASP A 469 ARG A 470 CYS A 471 LYS A 472 ASN A 473 LYS A 475 THR A 477 ASP A 479 SER A 503
006	000008.3810	00000000718862.0000	HIS A 394 THR A 395 HIS A 397 ARG A 418 PHE A 419 PHE A 467 ASP A 469 ARG A 470 CYS A 471 LYS A 472 ASN A 473 LYS A 475 THR A 477 ASP A 479 SER A 503
007	000008.3720	00000000729865.1875	PRO A 30 ALA A 31 PHE A 32 LEU A 33 CYS A 35 GLU A 36 LEU A 37 LYS A 38 PRO A 39 GLN A 779 THR A 780 VAL A 782 PRO A 783 GLY A 784 LEU A 785 ALA A 786
008	000008.3720	00000000729865.1875	PRO A 30 ALA A 31 PHE A 32 LEU A 33 CYS A 35 GLU A 36 LEU A 37 LYS A 38 PRO A 39 GLN A 779 THR A 780 VAL A 782 PRO A 783 GLY A 784 LEU A 785 ALA A 786

## Appendix B

### Appendix B.1

The organ coefficient is an important indicator for evaluating the degree of bacterial infection. *SE* infection leads to a significant decrease in the weight and food and water intake of mice, as well as pathological phenomena such as organ edema and congestion. Therefore, the increase in the organ coefficient was caused by *SE* infection. After the pretreatment with RA, the increase in the organ coefficient in the liver, kidney and spleen caused by *SE* was significantly reduced.

### Appendix B.2

RA regulated the gut microbiota in the *SE*-infected mice.

### Appendix B.3

The broad-spectrum antibiotic pretreatment significantly reduced the number of intestinal bacteria in the mice.
